# ROR1 Potentiates FGFR Signaling in Basal-Like Breast Cancer

**DOI:** 10.3390/cancers11050718

**Published:** 2019-05-24

**Authors:** Gaurav Pandey, Nicholas Borcherding, Ryan Kolb, Paige Kluz, Wei Li, Sonia Sugg, Jun Zhang, Dazhi A. Lai, Weizhou Zhang

**Affiliations:** 1 Department of Pathology, College of Medicine, University of Iowa, Iowa City, IA 52242, USA; gpandey@wustl.edu (G.P.); nicholas-borcherding@uiowa.edu (N.B.); paige-kluz@uiowa.edu (P.K.); 2 Cancer Biology Graduate Program, College of Medicine, University of Iowa, Iowa City, IA 52242, USA; 3 Medical Scientist Training Program, College of Medicine, University of Iowa, Iowa City, IA 52242, USA; 4 Department of Pathology, Immunology, and Laboratory Medicine, University of Florida, Gainesville, FL 32610, USA; ryankolb@ufl.edu (R.K.); weili1978@gmail.com (W.L.); 5 Department of Surgery, College of Medicine, University of Iowa, Iowa City, IA 52242, USA; sonia-sugg@uiowa.edu; 6 Division of Hematology, Oncology and Blood & Marrow Transplantation, Department of Internal Medicine, College of Medicine, University of Iowa, Iowa City, IA 52242, USA; jun-zhang-1@uiowa.edu; 7 Speed Biosystems, Gaithersburg, MD 20878, USA; alex.lai@speedbiosystems.com

**Keywords:** breast cancer, ROR1, FGFR signaling, cancer therapy

## Abstract

Among all breast cancer types, basal-like breast cancer (BLBC) represents an aggressive subtype that lacks targeted therapy. We and others have found that receptor tyrosine kinase-like orphan receptor 1 (ROR1) is overexpressed in BLBC and other types of cancer and that ROR1 is significantly correlated with patient prognosis. In addition, using primary patient-derived xenografts (PDXs) and *ROR1*-knockout BLBC cells, we found that ROR1^+^ cells form tumors in immunodeficient mice. We developed an anti-ROR1 immunotoxin and found that targeting ROR1 significantly kills ROR1^+^ cancer cells and slows down tumor growth in ROR1^+^ xenografts. Our bioinformatics analysis revealed that ROR1 expression is commonly associated with the activation of FGFR-mediated signaling pathway. Further biochemical analysis confirmed that ROR1 stabilized FGFR expression at the posttranslational level by preventing its degradation. CRISPR/Cas9-mediated *ROR1* knockout significantly reduced cancer cell invasion at cellular levels by lowering FGFR protein and consequent inactivation of AKT. Our results identified a novel signaling regulation from ROR1 to FGFR and further confirm that ROR1 is a potential therapeutic target for ROR1^+^ BLBC cells.

## 1. Introduction

Breast cancer is the most common cause of cancer-associated death among women worldwide [1] and the 2nd most lethal cancer among women in the United States [2]. Molecular heterogeneity in breast cancer results in sub-designations that are informative for prognosis, treatment response, and/or disease course [3]. Of these, approximately 15–20% are BLBC sharing 70–80% overlapping with clinical defined triple-negative breast cancer (TNBC) that lacks estrogen receptor, progesterone receptor, and HER2 [3,4,5]. BLBC represents one of the most aggressive subtypes of breast cancer and lacks targeted therapies, which underscores the need for further investigations [3,4]. Preclinical and clinical trials for targeted therapies in BLBC have focused on several targets with limited success. Investigations into growth factor signaling antagonists, like epidermal growth factor receptor (EGFR) pathway or vascular endothelial growth factor receptor (VEGF) signaling demonstrated no improvement in survival for patients [6,7]. More recently, poly-ADP-ribose polymerase (PARP) inhibitors have shown promise in BLBC patients with germline mutations in *BRCA1/2* [8]. Despite the promise of PARP inhibitors, *BRCA1/2* mutations account for around 15% of BLBC [9].

ROR1 is a type I transmembrane protein which is expressed during embryonic development and tumorigenesis [10], thus it has been described as an oncofetal protein [11]. Recent observation using a newly-developed antibody demonstrated ROR1 to be expressed in normal tissues such as the parathyroid gland, pancreatic islet, regions of esophagus, stomach, and duodenum [12]. ROR1 protein is overexpressed in several types of leukemia, but prominently in chronic lymphocytic leukemia (CLL), as well as a variety of solid malignancies including: breast, melanoma, pancreas, lung, ovary, colon, and renal cell carcinomas [13,14,15,16]. In breast cancer, ROR1 has been shown to promote cell proliferation, resistance to apoptosis, and epithelial-mesenchymal transition (EMT) [15,17,18]. The important role of ROR1 in cancer prompted early therapeutic investigations, including the development of anti-ROR1 antibodies [19], antibody-drug conjugates (antibody-fused to bacterial toxin) [20], chimeric antigen receptor (CAR) T cell therapy [21,22,23,24], as well as small molecule inhibitors [25]. Although certain normal tissues express ROR1 [12], targeting ROR1 in animal models including primates [24] appears to have very limited toxicity in preclinical studies and shows promise in the treatment of different types of cancer. An early clinical trial targeting ROR1 using a humanized antibody reported the therapy to be well tolerated in human CLL patients, but with limited improvement on disease progression [19]. Conversely, a recent meeting report has cast doubt on ROR1-targeted CAR-T therapy due to its lack of efficacy in reducing tumor burden and high pulmonary toxicity [21]; the field may become further clouded by a recent withdrawal of a clinical trial with unknown reason (clinicaltrials.gov identifier: NCT02194374). The question remains whether ROR1 can be targeted in solid cancers and what other ROR1-targeting methods can be used to increase efficacy and minimize toxicity.

FGFR is a promising target for different types of cancer and several FGFR-specific inhibitors are in clinical trials for various cancer types [26]. FGF signaling is a vital paracrine mediator of mammary gland formation and mammary stem cell maintenance [27]. *FGFRs* are amplified in 5–10% of breast cancers including TNBC [26,28,29]. Clinical development of FGFR inhibitors has seen compounds enter into phase II clinical trials in several cancers [26]. In breast cancer, preclinical studies showed that FGFR inhibition led to a reduction in BLBC tumor growth via the inhibition of FGFR-mediated downstream MAPK and AKT activation [30]. The efficacy of FGFR inhibitors in cancer therapy, however, is generally limited [26]. As genomic alterations of FGFRs have been used as the only guidance in clinical trials, one of the reasons could be the disconnection between genomic alteration (mainly amplification) and protein expression/activation.

In our study, we found that in BLBC, FGFR1 protein level is regulated by ROR1 expression, a process independent of sustaining caveolae as recently reported [31]. It appears that ROR1 prevents FGFR1 from degradation in BLBC cells. This pathway is critically involved in invasiveness and tumorigenic properties in BLBC.

## 2. Results

### 2.1. ROR1 Expression Is Correlated with Poor Overall Survival in Certain Cancers

**Figure 1:**
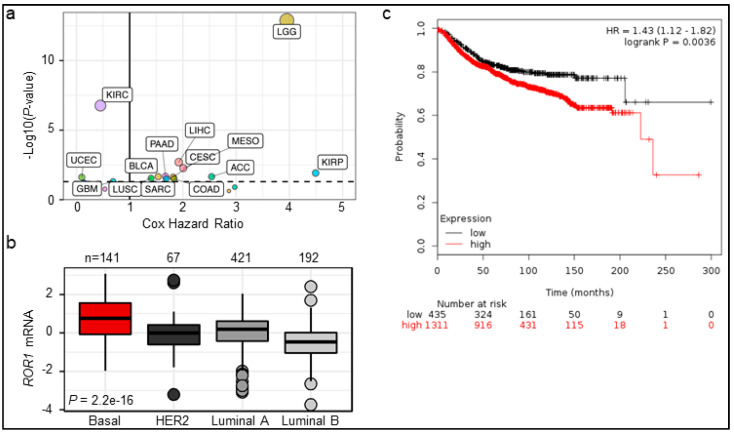
Higher expression of *ROR1* is correlated with poor overall survival in several cancers. (**a**) Cox proportional hazard regression summary of hazard ratios and –log10 (*p*-value) across the TCGA Pan Can cohort. Labeled points indicate significance of *p* < 0.05, which is also marked by the horizontal dotted line. (**b**) *Z*-score transformed mRNA for *ROR1* across PAM50 subtypes in the breast cancer TCGA cohort. Samples compared using one-way ANOVA test. (**c**) KM plotter was used to analyze *ROR1* expression and prognosis. Using *ROR1* max probe: 205805_s_at and optimal cutoff, *ROR1* mRNA is inversely correlated with distal metastasis free survival (DMFS) in all breast cancer patients (*n* = 1311 in the ROR1-high group and *n* = 435 in the low group, *p* = 0.0036).

Previous work has shown ROR1 expression in several cancers. To get a better view of *ROR1* expression in cancer, we thoroughly analyzed 29 types of cancer deposited in TCGA (Supplementary Figure S1). The highest *ROR1* expressers include mesothelioma (MESO), sarcoma (SARC), stomach adenocarcinoma (STAD), ovarian cystadenocarcinoma (OV), and pancreatic adenocarcinoma (PAAD). Our RNA results mirror similar results for ROR1 in IHC with elevated levels in pancreatic, ovarian, and lung cancers [12]. We also examined the prognostic value of *ROR1* across the TCGA, finding *ROR1* was a poor prognostic marker in 11 of 29 cancer types (Supplementary Figure S1a, red dots) and a good prognostic marker in 3 of 29 cancer types (Supplementary Figure S1a, blue dots). Additionally, we performed Cox proportional hazard regression analysis for *ROR1* mRNA in all 29 cancer types and found ROR1 has the worst prognosis in kidney papillary cell (KIRP) and low-grade glioma (LGG) with hazards ratios of 4.49 and 3.95, respectively (Figure 1a). Earlier reports have found ROR1 protein is expressed in TNBC and predicts poor prognosis in TNBC/BLBC [17]. Across all breast tumor samples in the TCGA, *ROR1* did not significantly predict survival with a hazard ratio of 1.33 (*p* = 0.14), but confirmed the highest *ROR1* expression in BLBC compared to other molecular subtypes (*n* = 821 with molecular subtype designations, Figure 1b). Due to the very limited survival information from TCGA breast cancer (around 15%), we utilized the widely-used KMplotter [32] and found that higher *ROR1* expression predicts shorter distal-metastasis-free survival (DMFS) in a total of 1746 breast cancer patients (Figure 1c, Affymetrix max probe for ROR1: 205805_s_at).

### 2.2. ROR1^+^ Cells Are Tumorigenic

**Figure 2:**
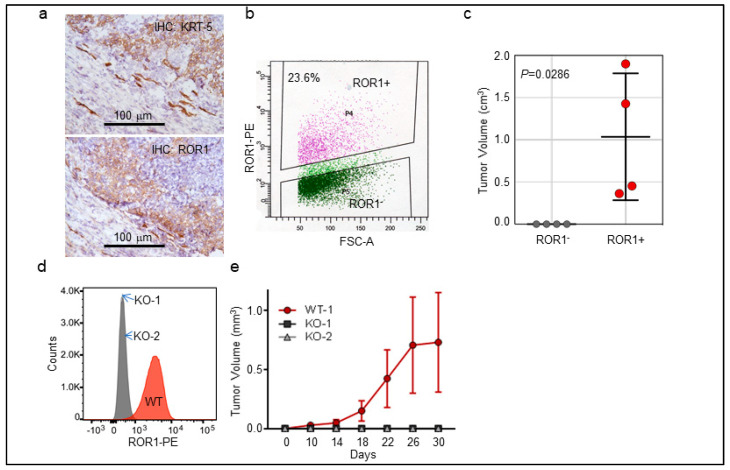
ROR1^+^ cells are tumorigenic. (**a**–**c**) One clinical TNBC specimen was stained with cytokeratin-5 (KRT-5) for defining basal cancer type and with ROR1 for its positivity (**a**). A fresh TNBC with confirmed basal type was digested into single cell suspensions, following with flow cytometry to separate into ROR1^+^ and ROR1^−^ epithelial (labeled by EpCAM+) populations (**b**). 5000 ROR1^+^ or ROR1^-^ TNBC cells were orthotopically injected into 7-week old immunodeficient female NSG mice for tumor growth, with one #4 fatpad injected with ROR1^+^ and the other #4 fatpad with ROR1^−^ cells for stringent comparison. Tumors were monitored (**c**, shown as mean ± SD, *n* = 4). (**d**,**e**) CRISPR/Cas9-mediated KO of ROR1 in MDA-MB-231 cells with two clones shown as negative of surface ROR1 staining by flow cytometry (**d**) and 2 million of WT and KO MDA-MB-231 cells were injected into #4 fatpad of NSG mice and tumor growth were measured (**e**, shown as mean ± SD, *n* = 7–8, *p* < 0.001, 2-way ANOVA).

To connect ROR1 function with human TNBC/BLBC, we collected several clinically defined TNBC cases and performed cytokeratin-5 (KRT-5) and ROR1 immunohistochemistry (IHC) (Figure 2a). We were able to establish a patient-derived xenograft model with confirmed basal phenotype based on positive KRT-5 expression (Figure 2a, top panel) with mosaic expression of ROR1 in cancer cells (Figure 2a, bottom panel). We purified ROR1^+^ and ROR1^-^ cancer cell population using flow cytometry (Figure 2b) and orthotopically injected 5000 cells into 7-week old female NOD/SCID/*Il2rg*^−/−^ (NSG mice, Jackson Laboratories, 005557). After a latency of 4 months, we found that only ROR1^+^ cells were able to form palpable tumors whereas ROR1^−^ cells did not develop any tumor up to 8 months (Figure 2c, *p* = 0.028). We used CRISPR/Cas9 system to knock out *ROR1* in MDA-MD-231 cells, a basal-B breast cancer cell line [33] with known ROR1 expression [17] and selected two stable clones with confirmed loss of membrane expression by flow cytometry (Figure 2d). We repeated this orthotopic injection using WT and KO cells of MDA-MB-231 cells in NSG mice and found that only WT cells were able to the grow tumors, while both *ROR1*-KO clones failed to grow tumors up to 3 months (Figure 2e, *p* < 0.001). These data provide support that ROR1^+^ cells are tumorigenic cells in the PDX model and established human cell lines.

### 2.3. Targeting ROR1+ Cells Using Immunotoxin in BLBC Therapy

**Figure 3:**
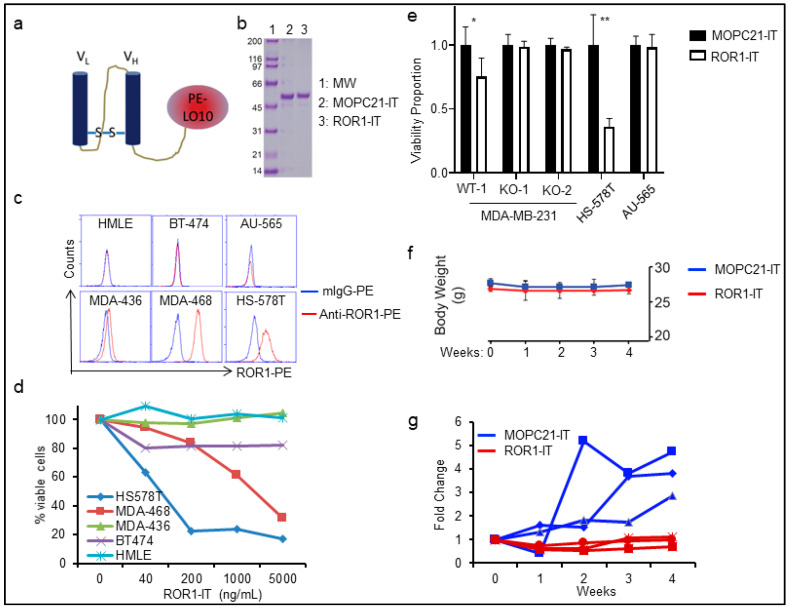
Targeting ROR1^+^ cancer cells with immunotoxin. (**a**,**b**) Construction and purification of anti-ROR1-immunotoxin by fusing variable region of anti-human ROR1 antibody (clone 2A2) with modified exotoxin A (PE_lo10_) from *Pseudomonas aeruginosa* (**a**), using variable region of MOPC21 (a non-specific mouse antibody)-fused with PE_lo10_ as control. Plasmids encoding ROR1-IT or MOPC21-IT were used to transform *E. coli* for purification (**b**). (**c**) A panel of breast epithelial or cancer cells were screened for ROR1 expression by flow cytometry, including immortalized HMLE cells, three ROR1-negative (BT-474 and AU-565) or low (MDA-MB-436), and two ROR1-positive (MDA-MB-468 and Hs578T) cells. (**d**) Cells in c. were treated with different doses of ROR1-IT or MOPC-21-IT (0, 40, 200, 1000, 5000 ng/mL). 48 h later, cells were collected and stained with 7-AAD for viability and quantitated by flow cytometry (*n* = 3, only data for ROR1-IT was shown here). (**e**) WT or KO cells of MDA-MB-231, ROR1^+^ Hs578T, or ROR1^−^ AU-565 cells were treated with 400 ng/mL of ROR1-IT or MOPC-21-IT for 48 h, following with counting of viable cells. ROR1-IT-treated cells were normalized to their corresponding MOPC-21-IT-treated cells (*n* = 3). * *p* < 0.05; ** *p* < 0.001. (**f**,**g**) ROR1^+^ Hs578T cells were injected orthotopically into NSG mice and treated with MOPC21-IT or ROR1-IT daily at 100 µg/injection for 4 weeks. Body weight (**f**) and tumor volume (**g**) were monitored weekly (*n* = 3).

The tumorigenic property of ROR1^+^ cells makes them ideal targets for therapy. As discussed in the introduction, many methodologies of targeting ROR1^+^ cells are being developed including antibodies, antibody-drug conjugates, CAR-T, and inhibitors. Antibody-drug conjugates have an advantage over naked antibodies for selective killing of target-positive cells even when the targets have relatively low levels of expression on cancer cells. Here we have developed a ROR1-targeting immunotoxin (ROR1-IT) comprising the variable region fragments (Fv) of an anti-ROR1 monoclonal antibody (clone 2A2) and the modified exotoxin of *Pseudomonas aeruginosa* (illustrated as in Figure 3a). We chose to use modified exotoxin PE_LO10_ that has most of its human B-cell epitopes mutated to eliminate immunogenicity in vivo while retaining its biological half-life and potent cellular toxicity [34,35]. As a control, we used anti-MOPC21-PE_lo10_ (MOPC21-IT) that does not target any antigen in human or mouse cells. The recombinant proteins were purified in bacterial inclusion body and re-folded (Figure 3b). To determine the specificity and toxicity of the ROR1-IT on cancer cells, we screened a panel breast cancer lines including ROR1-negative HMLE breast epithelial cells, HER2^+^ BT-474 and AU-565, and MDA-MB-436 cells, as well as ROR1^+^ MDA-MB-436 and Hs578T cells (Figure 3c). We found that ROR1-IT preferentially killed ROR1^+^ cells (Figure 3d); whereas MOPC21-IT did not significantly kill any cells even with the highest concentration used. We also treated WT or KO of MDA-MB-231 cells, together with ROR1+ Hs578T and ROR1- AU-565 cells, where ROR1-IT only killed ROR1^+^ Hs578T cells and WT MDA-MB-231 cells but not the two KO clones or ROR1^-^ AU-565 cells (Figure 3e). Daily i.v. infusion of MOPC21-IT (Figure 3f, blue line) or ROR1-IT (Figure 3f, red line) did not significantly influence body weight of tumor-bearing mice, but ROR1-IT completely halted tumor growth from HS-578T xenografts up to 3 weeks of treatment in NSG mice relative to control group (Figure 3g, *p* < 0.05, two-way ANOVA).

### 2.4. ROR1 Is Associated with FGFR Activation in Several Major Cancer Types

**Figure 4:**
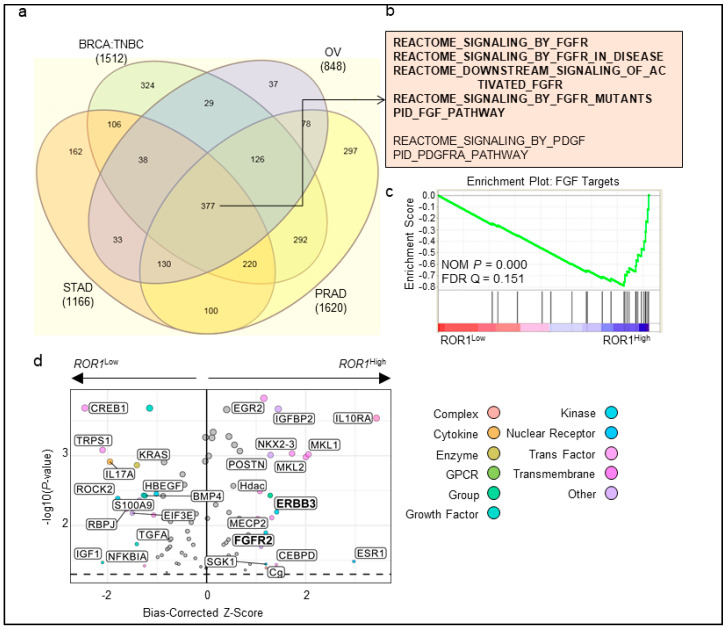
ROR1 is associated with FGFR signaling across different cancers. (**a**–**c**) Stomach adenocarcinoma (STAD: data from TCGA), triple-negative breast cancer (BrCa: data from GSE76275), ovarian (OV: data from TCGA) and prostate adenocarcinoma (PRAD: data from TCGA) were separated into low and high ROR1 expressing groups (77 samples with the lowest expression vs. 77 samples with the highest expression) and gene set enrichment analysis was performed. (**a**) Venn diagram of genesets significantly enriched (FDR < 0.25) in the ROR1 high group from each dataset. (**b**) Genesets related to FGFR and PDGFR receptor tyrosine kinase signaling pathways from the common 377 genesets in (**a**) are indicated. (**c**) Enrichment plot for FGFR geneset showing enrichment in ROR1-high expressing group in the BrCa TNBC dataset. (**d**) Ingenuity Pathway Analysis (IPA) for upstream regulators comparing the *ROR1*^high^ versus *ROR1*^low^ tertiles in the BLBC samples in the TCGA. Labeled regulators have a –log10 (*p*-value) > 1.3, the equivalent of *p* < 0.05, and bias-corrected *Z*-scores > 1.2.

ROR1 has been proposed to promote cancer progression via different mechanisms in a cancer type-dependent manner [13,17,18,31,36,37,38,39]. To determine if a common signaling pathway is associated with ROR1 expression, we downloaded several Affymetrix or RNA sequencing datasets for a thorough bioinformatic analysis among the top *ROR1* expressers, including TNBC of breast cancer, OV, PRAD, and STAD. Based on gene set enrichment analysis (GSEA) [40], we identified 1512 gene sets in TNBC, 848 in OV, 1166 in STAD, and 1620 in PRAD that are associated with *ROR1* expression with false discovery rates (FDR) less than 0.25 (Figure 4a). Among the common receptor tyrosine kinase (RTK)-relevant pathways, FGFR pathways are the most commonly enriched pathways in *ROR1*-high cancer specimens, following with platelet-derived growth factor receptor (PDGFR) (Figure 4b). The enrichment plot for FGF targets was shown to reflect activation of FGFR-pathways in TNBC (Figure 4c), as well as within lung adenocarcinoma (LUAD) in an independent analysis using the TCGA dataset (Supplementary Figure S1b, upper panel). PDGF pathway was also enriched in *ROR1*-high specimens (Supplementary Figure S1b, lower panel). We further analyzed TCGA BLBC breast cancer specimens using Ingenuity Pathway Analysis and found FGFR and ERBB3 RTK pathways are also enriched in ROR1-high BLBC specimens (Figure 4d). These data indicate that ROR1 may regulate FGFR-mediated signaling pathways.

### 2.5. ROR1 Stabilizes FGFR at the Post-Translational Level

**Figure 5:**
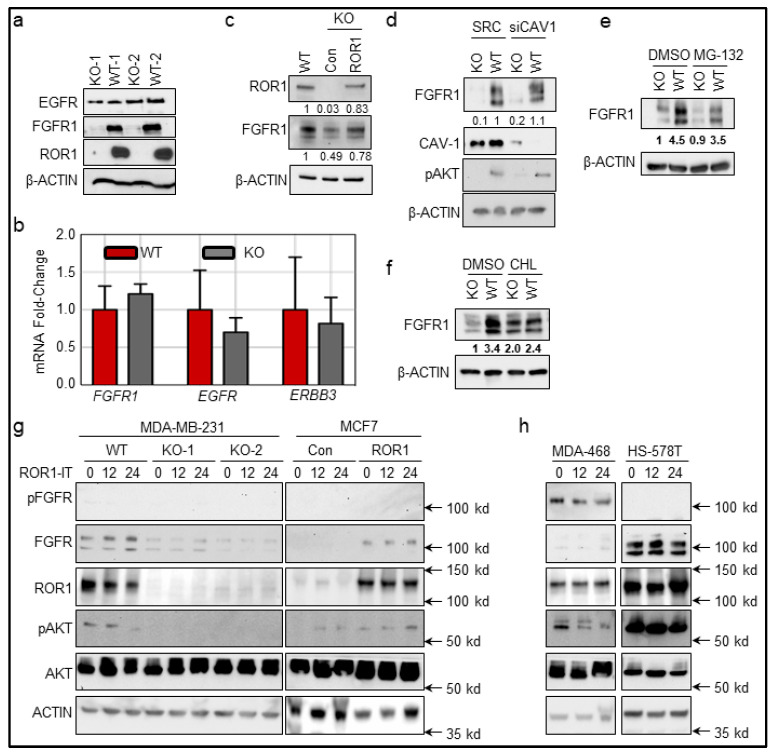
ROR1 stabilizes FGFR1 protein level. (**a**) CRISPR/Cas9-mediated KO of *ROR1* in MDA-MB-231 cells with two clones shown as ROR1-KO clones. Representative lanes are shown from immunoblots of cell lysate probed with the antibody against EGFR, FGFR1, ROR1, and β-ACTIN was used as loading control. (**b**) Real-time PCR analysis of *FGFR1*, *EGFR1*, and *ERBB3* (HER3) in MDA-MB-231 *ROR1*-WT or KO cells. (**c**) ROR1^−^ cells were transfected with *ROR1* plasmid for 48 h. Representative lanes are shown from immunoblots of cell lysate probed with the antibody against ROR1, FGFR1, and β-ACTIN was used as loading control. (**d**) Cells were transfected with scrambled control siRNA and Cav-1 (*CAV1*) siRNA for 48 h. Representative lanes are shown from immunoblots of cell lysate probed with the antibody against CAV-1, FGFR1, p-AKT and β-ACTIN was used as loading control. (**e**,**f**) Cells were treated with MG-132 (**e**) or chloroquine (**f**, CHL) for 4 h, or mock-treated with DMSO (**e**,**f**). Representative lanes are shown from immunoblots of cell lysate probed with the antibody against FGFR1 and β-ACTIN was used as loading control. For all western blots, summary of band densities, normalized to β-ACTIN (*n* = 3). (**g**,**h**) Listed cell lines were treated with 200 ng/mL of ROR1-IT or MOPC21-IT for the indicated periods. The 0 h control were lysates from MOPC21-IT-treated cells for 24 h. Cell lysates were collected in, following with SDS-PAGE and immunoblotting of pFGFR1, FGFR1, ROR1, pAKT, AKT and β-ACTIN was used as loading control. Con or ROR1 in MCF7 groups refers to MCF7 cells that stably express empty plasmid or ROR1-encoding plasmid. MDA-468 refers to MDA-MB-468 cells.

To study the role of ROR1 in FGFR signaling, we used our established two clones of MDA-MB-231 cells with *ROR1*-KO (Figure 5a). While we found that *ROR1*-KO did not alter EGFR expression, *ROR1*-KO resulted in loss of FGFR1 protein in both KO lysates (Figure 5a), but not mRNA expression of different RTKs (Figure 5b). To confirm that FGFR1 protein is directly mediated by ROR1 rather than some unknown secondary effect, we reconstituted ROR1 expression in the KO cells, which restored the FGFR1 protein (Figure 5c).

As ROR1 has been shown to mediate RTK activation via influencing caveolae function by stabilizing Caveolin-1 (CAV-1) [31], we silenced *CAV1*, a critical component in caveolae, and found that FGFR1 protein level was not altered by inhibition of caveolae in either WT or KO cells (Figure 5d). A similar result was observed when using MG-132, an inhibitor of proteasome (Figure 5e). These data exclude the potential involvement of caveolae and proteasome in FGFR1 degradation. A previous study suggested that endosome-lysosome pathways regulate the FGFR1 protein levels in the cells [41]. We treated cells with chloroquine, an inhibitor of lysosomal degradation, and found FGFR1 expression to be mostly restored in the *ROR1* KO cells (Figure 5f), indicating that loss of FGFR1 expression in the *ROR1*-KO cells is potentially via chloroquine-inhibitable pathways.

ROR1-IT kills ROR1^+^ cells mediated by exotoxin of *Pseudomonas aeruginosa*, via endocytosis and blocking protein translation [34,35]. Curiously, we determined the potential role of ROR1-IT on inhibiting ROR1/FGFR1-mediated signaling transduction. We used a collection of cells, including WT and KO cells of MDA-MB-231, MCF7 control or ROR1-expressing pooled cultures, or two ROR1^+^ MDA-MB-468 and Hs578T cells. We confirmed that ROR1 expression and AKT phosphorylation were correlated with FGFR1 expression in WT MDA-MB-231 cells (Figure 5g, MDA-MB-231 cells, comparing 0 h time points). ROR1 expression in MCF7 cells induced one of the isoforms of FGFR1 expression as well as AKT phosphorylation (Figure 5g, MCF7, 0 h time point). FGFR1 phosphorylation in those two cells was too low to be detected under these conditions and without ligand induction (Figure 5g), but readily detectable in MDA-MB-468 cells with lower FGFR1 expression (Figure 5h), suggesting that MDA-MB-468 may have an autocrine FGFR activation and produce its ligands. ROR1-IT treatment at 200 ng/mL concentration that led to partial killing of ROR1^+^ cells (Figure 3d) failed to consistently alter ROR1, FGFR1, pAKT or AKT levels among all ROR1^+^ cells (Figure 5g,h), suggesting that ROR1-IT did not kill cancer cells via signaling inhibition in all ROR1^+^ cancer cells.

### 2.6. ROR1 Promotes Invasion through FGFR1

**Figure 6:**
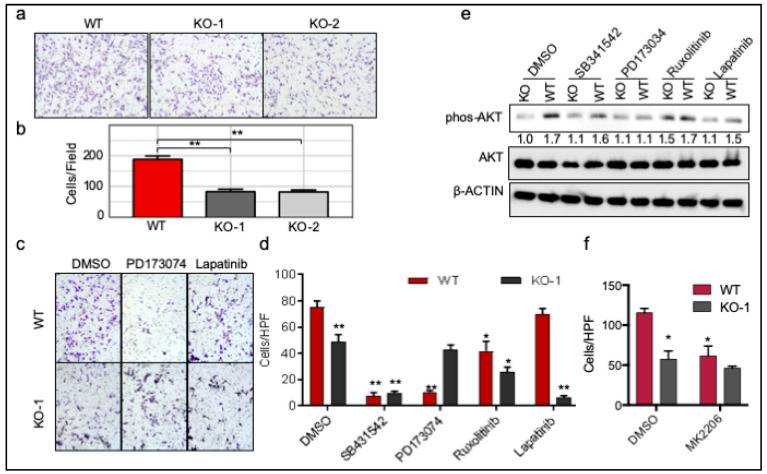
ROR1 regulates invasion through FGFR1 in BLBC cells. (**a**) Cells in Figure 5a were used for invasion assay. Scale bar represents 200 μm. (**b**) The graph represents the average cell number/microscopic field (*n* = 3). (**c**,**d**) WT or KO Cells were plated for invasion assay and treated with control DMSO, SB-431542 (5 µM), PD173074 (50 nM), ruxolitinib (10 µM), or lapatinib (100 nM) for 24 h. Representative images were shown (**c**) and quantitated (**d**, shown as mean ± SD, *n* = 3–4). (**e**) WT or KO Cells were treated with same set of inhibitors for 2 hours in the complete media. Cells lysates were collected for immunoblotting with pAKT and AKT, with β-ACTIN as a loading control. (**f**) WT or KO Cells were plated for invasion assay and treated with control DMSO or AKT inhibitor MK2206 for 24 h and quantitated. Data shown as mean ± SD (*n* = 3), * *p* < 0.05, ** *p* < 0.01.

To understand the function of ROR1 in BLBC, we performed studies to characterize the impact of ROR1-deficiency on cell growth, and invasion assays. We found that ROR1-deficiency led to a decrease in proliferative capacity (Supplementary Figure S2) and in invasion (Figure 6a and quantitated in Figure 6b), in agreement with previously published results [17]. To determine if FGFR-mediated pathway is critical for ROR1-induced invasion, we treated the MDA-MB-231 WT and *ROR1* KO cells with FGFR inhibitor PD173034, or using other EMT/invasion relevant kinase inhibitors such as SB-431542 (TGFβR1 inhibitor), ruxolitinib (JAK inhibitor), or lapatinib (EGFR family inhibitor) (Figure 6c,d). We found that FGFR inhibition specifically reduced ROR1-mediated invasion in the WT cells, but not in the KO cells (Figure 6c,d). Other kinase inhibitors, however, had similar effects on reducing invasion irrelevant of ROR1 status (SB-431542 and ruxolitinib), or preferentially inhibited invasion of KO cells (lapatinib) (Figure 6d). As AKT activation has been commonly linked to ROR1 function in different cancer types [14,15,17,39], we confirmed that ROR1-deficiency led to a significant decrease in AKT activation (Figure 6e, first two lanes). FGFR inhibition specifically reduced AKT phosphorylation in WT cells, but not in the KO cells (Figure 6e), correlating well with the invasion data (Figure 6c,d). As we and others have defined the role of AKT in EMT and invasion [42,43,44], AKT inhibition using MK2206 resulted in a significant decrease in invasion from WT cells, but not significantly in the KO cells (Figure 6f), suggesting that ROR1/FGFR/AKT axis is critically involved in mediating invasive capacity of ROR1^+^ cells. Analysis of TCGA breast cancer dataset also revealed that ROR1 expression is positively correlated with many gene sets involved in EMT and metastasis in BLBC (Supplementary Figure S3).

## 3. Discussion

BLBC remains an aggressive subtype of cancer with worse prognosis compared to luminal or HER2-enriched molecular subtypes of breast cancer. Targeted therapies for BLBC have mostly failed in clinical trials except for the relative success of PARP inhibitors in patients with *BRCA1/2* mutations (around 15% of total BLBC cases) [9]. Thus, the identification and development of new therapies for BLBC represent an unmet clinical need. Similar to other studies [15,17,18], we have found elevation of *ROR1* in BLBC compared to other molecular subtypes of breast cancer (Figure 1b), as well as other cancer types [13,14,15,16]. Our complete analysis using TCGA datasets further demonstrates that *ROR1* is expressed highly among several major cancer types (Supplementary Figure S1) and is a poor prognostic factor in 11 different cancer types (Supplementary Figure S1, Figure 1a). These data strongly indicate a potential common signaling pathway that can mediate the oncogenic process in relevance of ROR1 expression. Several papers have established the involvement of EGFR in ROR1-mediated survival signaling within carcinomas [31,39], which may not be able to explain why ROR1 is expressed the highest in the sarcoma and mesothelioma and predicts bad prognosis in these cancers (Supplementary Figure S1a). Using an unbiased bioinformatic approach, we identified FGFR pathway to be the common RTK pathway that is significantly correlated with *ROR1* expression in various cancer types. As both ROR1 and FGFR have been emerging targets for cancer therapy, it is essential to understand how these two molecules integrate during cancer progression. We believe that ROR1 stabilizes FGFR1 at protein level, likely via the chloroquine-inhibitable protein degradation pathway. Collectively, ROR1 and FGFR1 together lead to the activation of AKT pathway and cancer cell invasion. At a mechanistic level, we failed to detect physical interaction between ROR1 and FGFR1 via co-immunoprecipitation, either due to the transient nature of the interaction or an indirect mechanism involved in shuttling FGFR1 back to membrane for recycling rather than sending it to lysosome for degradation.

The FGFR pathway has been an intriguing target for cancer therapy due to genomic amplification, mutations, and fusions in several cancer types such as breast and lung cancers [26,28]. Several inhibitors have been undergoing clinical trials, most of which have ended up with unfavorable results in cancer patients with genetically altered FGFRs [26]. Most clinical trials used or have been using genetic information as the only method to determine patient recruitment [26]. It is perceivable that FGFR inhibitors will not work in cancers without functional proteins. As ROR1 expression stabilizes FGFR protein (Figure 5) and is significantly associated with FGFR pathway activation in various cancer types (Figure 4), ROR1 protein expression may be considered as a surrogate marker for FGFR activation among carriers with genetic FGFR alterations. Our results together suggested that FGFR1 might be a therapeutic target in ROR1^+^ TNBC. In fact, preclinical studies did observe BLBC cell lines were more sensitive to FGFR1 inhibitor PD173074 (same compound we used here) [45]. The proposed FGF2 ligand expression despite helpful, was later considered not ideal, along with proposed biomarkers in other studies, to predict the response to FGFR inhibition [46]. This is reflected in early phase clinical trials using FGFR inhibitors—in which the overall effect is modest, and identification of the sensitive subpopulation remains challenging [26]. Along the same line, a recent paper using a proteomic strategy identified another protein, Protein Tyrosine Phosphatase Receptor Type G (PTPRG), to be involved in controlling FGFR1 activity and influences sensitivity of sarcoma cells to FGFR kinase inhibitors [47]. In order to maximize the efficacy of FGFR inhibitors in cancer patients, the field demands a better way to identify patient populations using methods such as IHC staining or flow cytometry, in addition to the *FGFR* genetic information.

In addition to stabilizing FGFR1 protein, ROR1 has clear roles in other RTK pathways [18,31,39] and non-canonical WNT signaling transduction in a cancer type- and context-dependent manner. We have identified that PDGFR-mediated pathway is also commonly associated with ROR1 expression in various cancer types (Figure 4). Since IMC-3G3, a monoclonal antibody against PDGFRα, has been approved by FDA in treating sarcoma patients [48], it will be interesting to know if ROR1-positive cancer cells are more sensitive to PDGFR inhibition. 

At biological level, we found that ROR1 is critically involved in invasion of BLBC cells (Figure 6) and tumorigenicity in our newly-established PDX model (Figure 2), proving its critical role in BLBC pathogenesis. The lack of outgrowth of tumors of the ROR1-deficient cells suggests an inhibition or lack of tumor-initiating cells, implicating the potential of ROR1 therapies to specifically target relapse potential, a major complication of BLBC. We strongly believe ROR1 is an outstanding target for BLBC and hence developed an immunotoxin to target ROR1^+^ cancers. We chose this antibody-drug conjugate due to several concerns: (1) Efficacy: naked antibody-mediated cancer therapy requires very high surface concentration, such as Herceptin for HER2^+^ cancers and cirmtuzumab for ROR1 in CLL and mantle cell lymphoma. ROR1 in BLBC or other solid cancers has variable expression levels (as exampled by Figure 5b where continuous ROR1 expression was observed). Immunotoxins require much lower surface receptor for killing due to the toxic effect of bacteria toxin on target cells (i.e., ROR1^+^ cells). (2) Toxicity: clinical trials using PE38, an earlier version of PE_Lo10_, have been published in several cancer types with very favorable impact on cancer patients and manageable toxicity. (3) Biological half-life: The lower immunogenicity of PE_Lo10_ warrants longer biological working time as it limits antibody production against this bacterial toxin protein. It is however unavoidable that some patients may develop neutralizing antibody. Other means of antibody-drug conjugate should be also taken into consideration during clinical development. The efficacy of small molecule-based ROR1 inhibitor or CAR-T therapy in solid cancer is yet unknown. The field has been arguing about the kinase activity of ROR1 as its kinase domain has several key amino acids different from those within a classic kinase domain, but residual auto-phosphorylation has been shown in purified proteins and phosphorylation of ROR1 can also be identified [25,49]. A kinase inhibitor has recently been developed with impressive efficacy in inducing apoptosis of ROR1^+^ CLL cells [25,49]. CAR-T strategies targeting ROR1^+^ cancer cells have also been developed and shown to have limited toxicity in primates and should be efficacious in eradicating ROR1^+^ CLL [24], but CAR-T efficacy in solid cancers generally has yet to be established. Underscoring the clinical application of therapies against ROR1 are two early-phase clinical trials into anti-ROR1 antibodies (NCT02776917) and ROR1-directed CAR T cell therapy (NCT02706392) that are recruiting patients with metastatic breast cancer. In summary, ROR1 seems to be an outstanding target for cancer therapy or to be used as a surrogate marker for redirecting patient populations for existing therapeutics such as FGFR inhibitors or potentially PDGFR antibody-based therapeutics.

## 4. Materials and Methods

### 4.1. TCGA Analysis

Log2-transformed RNA sequencing data by expectation maximization values for the breast cancer (BRCA) and PANCAN TCGA datasets were downloaded from the Memorial Sloan Kettering cBioPortal [50]. Samples with the categorical PAM50 designations based on the TCGA Analysis Working Group were isolated and scaled into *Z*-scores. Survival calculations were performed in R (v3.5.1) using the survival (v2.42-6) and survminer (v0.4.0) R packages [51]. Optimal cut-off for the basal-like breast cancer in the TCGA data utilized the surv_cutpoint function of the survminer package and the cut-off was determined by log-rank testing.

### 4.2. Pathway Analysis

Gene set variation analysis (GSVA) was performed for all the basal-like breast cancer samples in the TCGA, finding enrichment scores across the MSigDB C2.all.v6.1 gene sets [40]. Invasive/metastatic/EMT-related gene sets were isolated using text mining and visualized based on *ROR1* mRNA expression using the Complex Heatmap R package (v3.6) [51]. Further pathway analysis was performed using Ingenuity Pathway Analysis (Qiagen, Hilden, Germany), isolating predicted upstream regulators based on *ROR1*^high^ versus *ROR1*^low^ tertile differential gene expression comparison in basal-like breast cancer. Upstream regulators results were displayed based on –log10 (*p*-value) of the overlap of genes in the pathway and the predicted bias-corrected *Z*-score of change in the *ROR1*^high^ basal-like breast cancer samples. Labeled regulators have a –log10 (*p*-value) > 1.3, the equivalent of *p* < 0.05, and bias-corrected *Z*-scores > |1.2|.

### 4.3. Cell Culture and Transfection

MDA-MB-231 (ROR1^+^), MDA-MB-436(ROR1^−^), MDA-MB-468(ROR1^+^), BT-474(ROR1^−^), Hs578T(ROR1^+^), HMLE(ROR1^−^), and HEK293T cell were all obtained from the American Type Culture Collection (ATCC, Manassas, VA, USA). Breast cancer cells were maintained in Dulbecco’s Modified Eagle Medium (DMEM, Invitrogen, Carlsbad, CA, USA) supplemented with 10% fetal bovine serum with 0.1% bovine pancreas insulin (Sigma-Aldrich, St. Louis, MO, USA). HMLE cells were cultured using full MEBM (Lonza, Basel, Switzerland) complemented with 10% FBS (Sigma-Aldrich), 100 U/mL of penicillin-streptomycin, 2 mM of L-glutamine, 10 ng/mL of human epidermal growth factor (EGF), 0.5 µg/mL of hydrocortisone and 10 µg/mL of insulin (Lonza). HEK293T were cultured with DMEM with 10% fetal bovine serum. Cells were all grown at 37 °C in 5% CO_2_ in a humidified incubator. For transfection, MDA-MB-231 or MCF7 cells were transfected with empty pEZ-m13 or pEZ-m13-ROR1 plasmid (GeneCopoeia, Rockville, MD, USA) by using GeneTran III (Biomiga, San Diego, CA, USA) transfection reagent. Stable cells were generated by selection of transfected cells with 800 µg/mL or 400 µg/mL of G418, respectively (Thermo Fisher Scientific, Waltham, MA, USA). For cell growth assay, cells were plated in 6 well plates at 5 × 10^4^ cells/well and maintained at 37 °C in a humidified incubator. Cells were counted using the Neuberger chamber after 48, 72, and 96 h.

### 4.4. Construction and Purification of Immunotoxin

Immunotoxin targeting ROR1 was essentially prepared as previously published [20], except that toxin PE38 was replaced with PE-LR-LO10 [34].

### 4.5. In Vitro Invasion Assay

Transwell invasion assays were performed. Briefly, transwell insert (pore size 8 µm, Fisher Scientific, Waltham, MA, USA) were coated (for invasion) with 10% growth factor reduced matrigel (Corning, Corning, NY, USA). Cells were trypsinized and resuspended into serum-free medium. 5 × 10^4^ cells in 0.5 mL medium were reseeded into the upper chamber with or without inhibitors, and 0.5ml medium with 5% FBS was added to the lower chamber as chemoattractant. After 24 h incubation, cells on the upper surface of the insert were removed with cotton swab. The bottom cells were fixed with 4% para-formaldehyde and stained with 0.1% crystal violet (Sigma-Aldrich) for 30 min at room temperature. The number of invading cells was counted from 5 randomly selected fields.

### 4.6. Flow Cytometry

Cells were suspended in PBS with 2% fetal bovine serum (FACS buffer) and incubated with APC conjugated anti-ROR1 antibody (BioLegend, San Diego, CA, USA) for 30 min on ice. Cells were then washed twice with FACS buffer. Cells were sorted by flow cytometry using a BD FACS Canto II (BD Bioscience, San Joes, CA, USA) and analyzed using Flowjo software (FlowJo, Ashland, OR, USA).

### 4.7. CRISPR/Cas9

LentiCRISPRv2 (Plasmid #52961, Addgene, Cambridge, MA, USA) system was used to KO the *ROR1* gene [52]. The guide RNA (gRNA) for human *ROR1* was 5′-CACCGATCGCGCTCGCGGCATCCAG-3′. The lentiCRISPRv2-gRNA sequence was ligated using Roche repaid ligation Kit (Roche Holdings, Basel, Switzerland). The HEK293T cells transfected with 6 µg of the lentiCRISPR2-gRNA plasmid, 3 µg of pMD2-VSVG plasmid and 3 µg of the ∆CMV using GeneTran III (Biomiga, San Diego, CA, USA). The lentiviral stock was then collected after three days and centrifuged at 3000 rpm for 10 min at 4 °C to remove the cell debris. The supernatant was filtered through 0.45 µm filter, aliquoted and stored at −80 °C. MDA-MB-231 cells were transduced with lentiviral vector. 48 h post transduction, the cells were then selected with 1 µg/mL of puromycin for 4 days. The stable KO clones were confirmed using flow cytometry.

### 4.8. Western Blotting 

Cells were lysed in cell lysis buffer (50 mM Tris-HCl pH 7.5, 1 mM EDTA, 1 mM EGTA, 1% Triton X-100, 100 mM KCl, 50 mM NaF, 1 mM Na3VO4, 100 nM aprotinin, 1 µM bestatin, 1 mM *p*-amidinophenylmethanesulfonyl fluoride (PMSF)). Protein lysates (30 μg) were separated by SDS-PAGE and transferred onto PVDF membrane (Bio-Rad, Hercules, CA, USA). Membranes were immunoblotted with the respective antibodies as follows: anti-ROR1 (1:1000), anti-pAKT (1:1000), anti-FGFR1 (1:1000), β-ACTIN (1:5000), anti-AKT (1:1000), anti-EGFR (1:1000) from Cell Signaling (Cell Signaling Technologies, Danvers, MA, USA) and CAV-1 (1:1000) from Santa Cruz (Santa Cruz Biotechnologies, Dallas, TX, USA). Immunoreactive bands were detected using a chemiluminescence based detection system (Bio-Rad).

### 4.9. Real Time PCR 

Total RNA was isolated from cells using RNeasy Plus MiniKit (Qiagen) and cDNA was generated using Superscript III First Stand cDNA synthesis Kit (Thermo Fisher Scientific). mRNA expression levels of the respective genes of interest were quantified by real-time PCR using fast SYBR premix Ex Taq II (Takara, Mountain View, CA, USA) and analyzed using ViiA7 real-time PCR system (Thermo Fisher Scientific). Fold change in gene expression was calculated by using the 2^−∆∆CT^ method. The following primers were used: *EGFR1*, Forward: 5′CAT GGC AGG TAC AGT AGG ATA AG3′; reverse: 5′ACAGGGCACACACAGATTAG3′. *FGFR1*, forward: 5′ TCAGATGCTCTCCCCTCCTC3′; reverse: 5′CTACGGGCATACGGTTTGGT3′. *ERBB3*, forward: 5′CGAGATTCTGTCAGGGGGTG3′; reverse: 5′CATCTCGGTCCCTCACGATG3′.

### 4.10. Immunohistochemistry

Formalin-fixed paraffin-embedded breast cancer tissues were deparaffinized with xylene, rehydrated in ethanol, and H_2_O. Antigen retrieval was performed using citrate buffer pH 6.0 with 30 min steaming followed by cooling for 20 min at room temperature. After unmasking, slides were blocked and treated with primary antibodies anti-ROR1 (1:200, clone 6D2, kindly provided by Dr. Riddell [12]) and anti-KRT5 (1:100, clone poly19055, BioLegend) incubated for 3 h at room temperature. Slides were then incubated with Dako Rabbit Envision for 30 min (Dako-Aligent, Santa Clara, CA, USA). Next, slides were incubated with DAB-3,3′-diaminobenzidin. Sections were counter stained with hematoxylin and mounted in mounting medium.

### 4.11. Patient-Derived Xenografts Model

TNBC specimens were provided from tissue procurement core of Pathology, University of Iowa, under approved IRB protocol: 201003791. De-identified fresh TNBC specimens were delivered to the laboratory and processed into single cells that were stained with ROR1 and other epithelial marker EpCAM, following with flow cytometry of EpCAM^+^ROR1^+^ and EpCAM^+^ROR1^−^ cells. 5000 of each single cell populations were paired injected into the opposite #4 fatpads of 7-week old female NSG mice (*n* = 4). Tumor growth were followed and monitored for 6 month and final tumor size was calculated and compared.

## 5. Conclusions

ROR1 is a valid therapeutic target for basal-like breast cancer and likely other cancers. In particular, we identified a novel regulatory role of ROR1 in FGFR signaling transduction by maintaining protein stability of FGFR1. This interaction is important in regulating cancer cell invasion likely via downstream activation of AKT pathway.

## Supplementary Materials

The following are available online at https://www.mdpi.com/2072-6694/11/5/718/s1, Figure S1: Supplementary Figures to Figure 1 and Figure 4, Figure S2: ROR1 promotes cell growth in MDA-MB-231 cells, Figure S3: ROR1 expression is correlated with metastasis and EMT genesets in BLBC.
