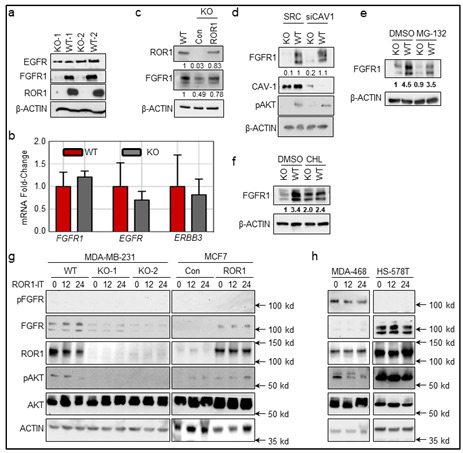# Correction: Pandey et al. ROR1 Potentiates FGFR Signaling in Basal-Like Breast Cancer. *Cancers* 2019, *11*, 718

**DOI:** 10.3390/cancers14184529

**Published:** 2022-09-19

**Authors:** Gaurav Pandey, Nicholas Borcherding, Ryan Kolb, Paige Kluz, Wei Li, Sonia Sugg, Jun Zhang, Dazhi A. Lai, Weizhou Zhang

**Affiliations:** 1 Department of Pathology, College of Medicine, University of Iowa, Iowa City, IA 52242, USA; 2 Cancer Biology Graduate Program, College of Medicine, University of Iowa, Iowa City, IA 52242, USA; 3 Medical Scientist Training Program, College of Medicine, University of Iowa, Iowa City, IA 52242, USA; 4 Department of Pathology, Immunology, and Laboratory Medicine, University of Florida, Gainesville, FL 32610, USA; 5 Department of Surgery, College of Medicine, University of Iowa, Iowa City, IA 52242, USA; 6 Division of Hematology, Oncology and Blood & Marrow Transplantation, Department of Internal Medicine, College of Medicine, University of Iowa, Iowa City, IA 52242, USA; 7 Speed Biosystems, Gaithersburg, MD 20878, USA

## Error in Figure

In the original publication [1], there was a mistake in Figure 5g,h as published. The authors made an unintentionally wrong cropping for Figure 5g,h for AKT blots. The corrected Figure 5, appears below. The authors apologize for any inconvenience caused and state that the scientific conclusions are unaffected. This correction was approved by the Academic Editor. The original publication has also been updated.